# Engineering the oleaginous yeast *Yarrowia lipolytica* for production of α-farnesene

**DOI:** 10.1186/s13068-019-1636-z

**Published:** 2019-12-23

**Authors:** Yinghang Liu, Xin Jiang, Zhiyong Cui, Zhaoxuan Wang, Qingsheng Qi, Jin Hou

**Affiliations:** 1 State Key Laboratory of Microbial Technology, Shandong University, Binhai Road 72, Qingdao, 266237 People’s Republic of China; 2 CAS Key Lab of Biobased Materials, Qingdao Institute of Bioenergy and Bioprocess Technology, Chinese Academy of Sciences, Qingdao, 266101 People’s Republic of China

**Keywords:** α-Farnesene, Mevalonate, *Yarrowia lipolytica*, Non-homologous end-joining

## Abstract

**Background:**

*Yarrowia lipolytica*, a non-traditional oil yeast, has been widely used as a platform for lipid production. However, the production of other chemicals such as terpenoids in engineered *Y. lipolytica* is still low. α-Farnesene, a sesquiterpene, can be used in medicine, bioenergy and other fields, and has very high economic value. Here, we used α-farnesene as an example to explore the potential of *Y. lipolytica* for terpenoid production.

**Results:**

We constructed libraries of strains overexpressing mevalonate pathway and α-farnesene synthase genes by non-homologous end-joining (NHEJ) mediated integration into the *Y. lipolytica* chromosome. First, a mevalonate overproduction strain was selected by overexpressing relevant genes and changing the cofactor specificity. Based on this strain, the downstream α-farnesene synthesis pathway was overexpressed by iterative integration. Culture conditions were also optimized. A strain that produced 25.55 g/L α-farnesene was obtained. This is the highest terpenoid titer reported in *Y. lipolytica*.

**Conclusions:**

*Yarrowia lipolytica* is a potentially valuable species for terpenoid production, and NHEJ-mediated modular integration is effective for expression library construction and screening of high-producer strains.

## Background

*Yarrowia lipolytica*, as the oleaginous yeast, is emerging as a model non-conventional oleaginous yeast [1]. It has been widely recognized as a valuable host for the production of lipid-based biofuels and oleo chemicals and has “generally regarded as safe” (GRAS) status [2, 3]. *Y. lipolytica* has the ability to utilize hydrophobic substrates and other cheap renewable carbon sources, and it can grow at a wide range of pH and salinity [4, 5]. Genome engineering tools have been developed to facilitate gene modification in *Y. lipolytica* [1, 6]. More importantly, *Y. lipolytica* has high metabolic flux of acetyl-CoA, making it an ideal cell factory for the synthesis of acetyl-CoA derived products, such as terpenoids or polyketides [7, 8]. For example, Markham et al. demonstrated that *Y. lipolytica* can produce a type III polyketide triacetic acid lactone (TAL) with a titer of 35.9 g/L, and a previously uncharacterized pyruvate bypass pathway was identified as a key pathway to increase TAL production [9]. Liu et al. demonstrated that the acetate uptake pathway in *Y. lipolytica* could function as an acetyl-CoA shortcut to achieve metabolic optimality in producing polyketides; the acetate-to-TAL conversion ratio (0.149 g/g) reached 31.9% of the theoretical maximum yield [10]. Yu et al. increased TAL titer by using nitrogen-limited growth conditions and multiple copies of the 2-pyrone synthase gene and converted TAL at 96% yield to pogostone, a valuable antibiotic [11]. Asides from polyketide, terpenoids are also synthesized using acetyl-CoA as a precursor. They belong to a large and diverse family with important medical and industrial properties, such as potent anticancer and antiviral activities and flavor/aroma properties. Many terpenoids are commercially valuable compounds. For example, artemisinin is a sesquiterpene endoperoxide with effective antimalarial properties [12], squalane (formed by catalytic hydrogenation of squalene) is a valuable cosmetic oil [13], and isopentadiene is used to produce various types of rubber [14]. *Y. lipolytica* has been engineered to synthesize many terpenoids, such as limonene, linalool, α-santalene, squalene, ginsenoside compound K, β-carotene, lycopene, and astaxanthin [15]. However, aside from carotenoids, the production of most of these compounds is still relative low, and only milligram has been produced [13].

Farnesene belongs to the sesquiterpene family of terpenoids, which have great economic value in medicine, cosmetics, condiments and bioenergy [16–18]. Furthermore, because of its high cetane number, low greenhouse gas emissions and good cryogenic properties, farnesene has been identified as an important substitute for jet fuel [13]. It is one of the simplest acyclic compounds produced by plants [16, 19]. However, its natural synthesis is severely restricted by plant growth, and fails to meet market requirements [15, 20]. Therefore, efforts have been made to engineer microbial cell factories for farnesene production. Farnesene is synthesized from precursor farnesyl pyrophosphate (FPP), which is produced via the mevalonate (MVA) pathway or the 2-C-methyl-d-erythritol-4-phosphate pathway. The MVA pathway is considered the more effective pathway for application in microbial metabolic engineering [7, 21]. The key enzymes of this pathway are HMG-CoA reductase (HMGR) and isopentenyl diphosphate isomerase (IDI). HMGR is an NADPH-dependent reductase in yeast and regarded as the rate-limiting enzyme of this pathway [22, 23].

Recently, heterologous production of farnesene has been performed based on microbial metabolic engineering and several hosts, including *Escherichia coli*, *Saccharomyces cerevisiae* and *Y. lipolytica*, have been engineered to produce farnesene [24–29]. You et al. constructed a β-farnesene producing strain of *E. coli* and overexpressed IDI and farnesyl diphosphate synthase (FPPS) to minimize isopentenyl diphosphate (IPP) accumulation. The titer of the final strain reached 8.74 g/L using glycerol as carbon source [28]. In *S. cerevisiae*, Meadows et al. rewired central carbon metabolism with four non-native metabolic reactions, enabling biosynthesis of cytosolic acetyl-CoA with a reduced ATP requirement, reduced loss of carbon to CO_2_-emitting reactions, and improved pathway redox balance. The strain could produce 25% more β-farnesene while requiring 75% less oxygen [29]. Farnesene reached a very high titer (130 g/L) and yield (0.173 g/g) in *S. cerevisiae*. In *Y. lipolytica*, overexpression of *tHMG1* (a truncated HMG-CoA reductase encoding gene), *IDI*, *ERG20* (an FPP synthase encoding gene) and codon-optimized α-farnesene synthase genes by integration into the genome resulted in the production of 259.98 mg/L α-farnesene [30]. However, the α-farnesene titer so far achieved in *Y. lipolytica* is much lower than that in *E. coli* or *S. cerevisiae*. Therefore, in this study, we chose α-farnesene as an example to explore the potential of *Y. lipolytica* to produce terpenoids.

Although several genome engineering tools have been developed in *Y. lipolytica*, low homologous recombination (HR) efficiency limits the efficiency of HR-based targeted integration. Non-homologous end-joining (NHEJ) is the predominate repair pathway for DNA double-strand breaks (DSB) in *Y. lipolytica* [31, 32]. We have recently reported that NHEJ-mediated genome integration in *Y. lipolytica* generates variation in the chromosomal locations of the inserted fragments and in gene copy numbers, resulting in expression differences of the integrated genes or pathways. This can be used to create gene expression libraries by one-step integration, which can be applied for enzyme production and biosynthetic pathway optimization [33]. This method facilitates pathway optimization and high-producer selection. In this study, using this method, we engineered *Y. lipolytica* for efficient α-farnesene production, and further improved the titer through optimization of the fermentation conditions. We achieve the highest terpenoid production reported in *Y. lipolytica*. Our work demonstrates the potential of *Y. lipolytica* to produce terpenoids.

## Results and discussion

### Construction of high mevalonate production strains through NHEJ-mediated genome integration

**Fig. 1:**
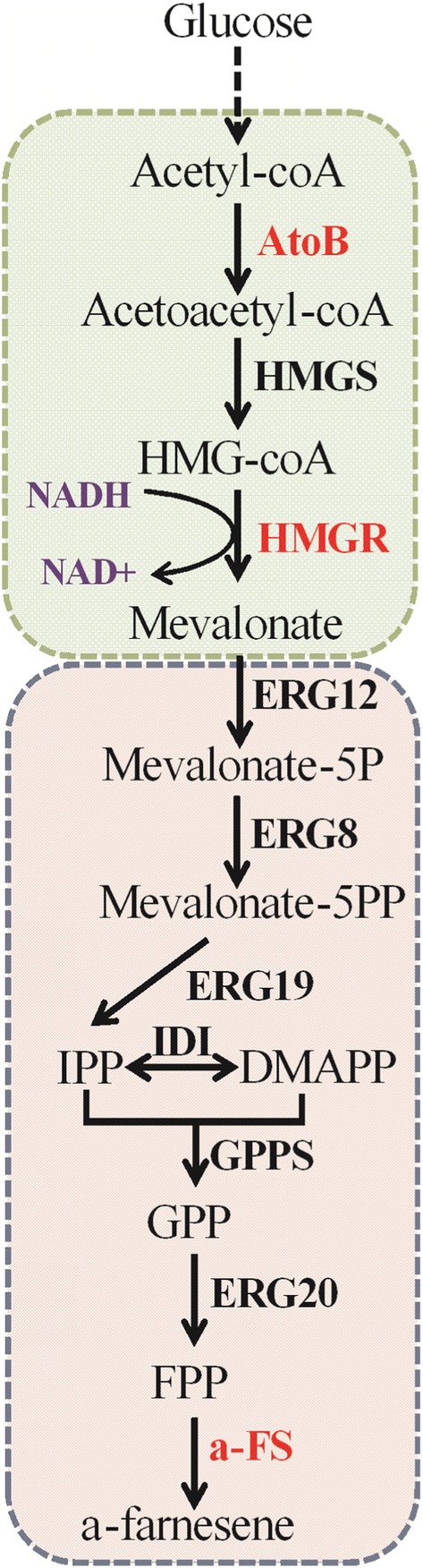
Metabolic pathway of α-farnesene synthesis from glucose in *Yarrowia lipolytica*. In the first module, acetyl-CoA is converted to mevalonate, providing precursor for the second module. In the second module, mevalonate is converted to α-farnesene. Red bold font, expression of heterologous proteins. Black bold font, overexpression of endogenous proteins. AtoB, acetyl-CoA acetyltransferase from *Escherichia coli*; HMGR, NADH-dependent HMG-CoA reductase from *Bordetella petrii*; α-FS, α-farnesene synthetase from apple (*Malus *×* domestica*)

**Fig. 2:**
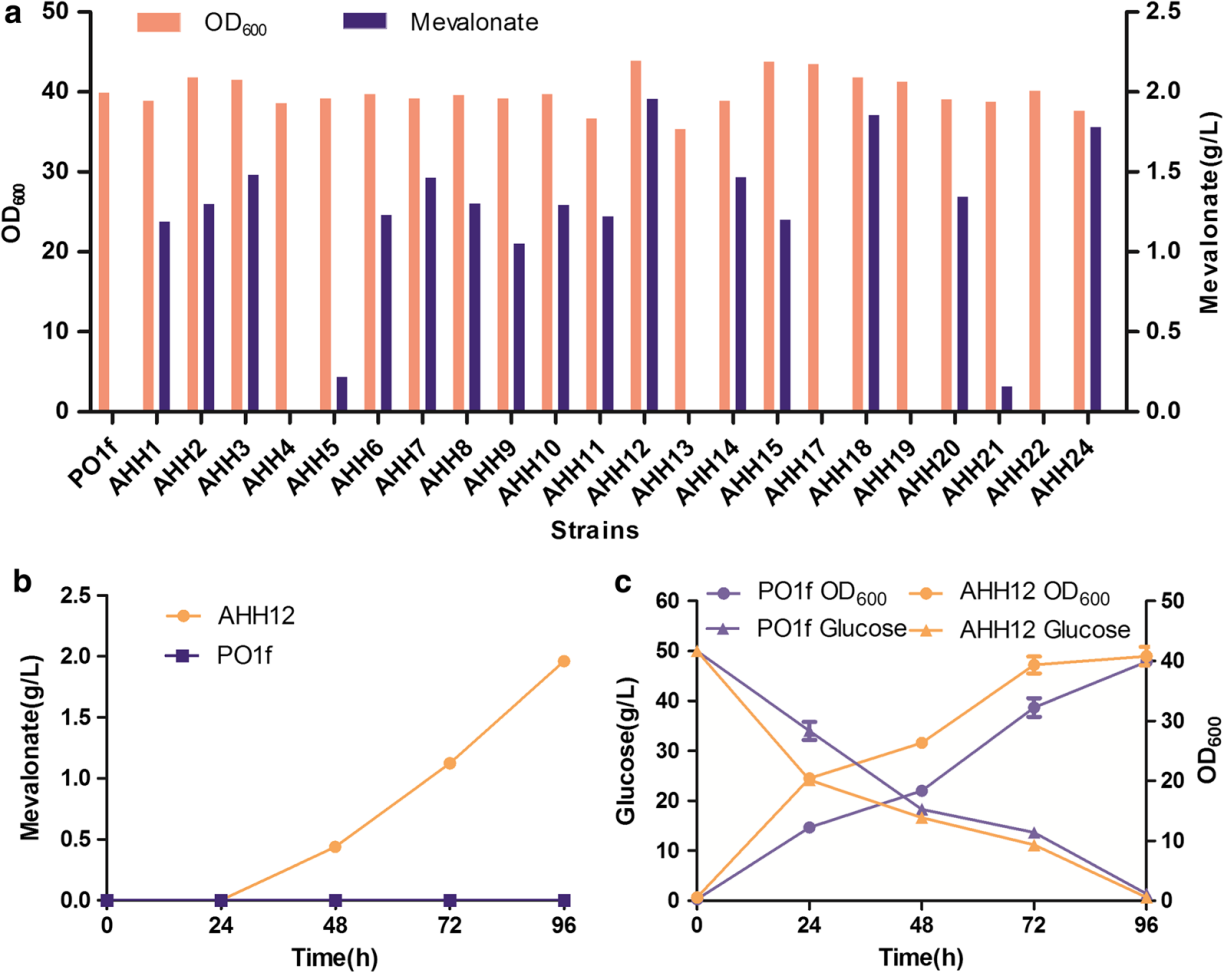
Screening of mevalonate production strain. **a** Mevalonate production and OD_600_ of different transformants with randomly integrated fragment containing *AtoB*, *HMGS* and *HMGR* were compared at 96 h. **b** Mevalonate accumulation curve of strains AHH12 and PO1f (control) during fermentation. **c** Growth and glucose consumption of strains AHH12 and PO1f during fermentation. All strains were grown in 300-mL shaken flasks containing 50 mL YPD medium and were cultured at 30 °C, 220 rpm. Data represent the mean ± SD of biological triplicates in **b** and **c**

α-Farnesene is synthesized using FPP as precursor, which is the intermediate product of the MVA pathway. To provide sufficient precursor for the synthesis of α-farnesene and to direct flux from acetyl-CoA to MVA synthesis, we first constructed a strain with high MVA production. MVA is synthesized from acetyl-CoA through the action of three enzymes—acetyl-CoA acetyltransferase (ERG10/AtoB), HMG-CoA synthase (ERG13/HMGS), and HMG-CoA reductase (HMGR) (Fig. 1). It is reported that AtoB, acetyl-CoA acetyltransferase from *E. coli*, has better catalytic activity in *S. cerevisiae*. In addition, the cofactor NADH is generally more abundant than NADPH in *Y. lipolytica*, and the use of NADH-dependent HMGR is reported to be more conducive for the production of farnesene [29]. Therefore, genes encoding *Y. lipolytica*-codon optimized AtoB and NADH-dependent HMGR from *Bordetella petrii*, and endogenous HMGS from *Y. lipolytica*, were inserted into the same plasmid under the control of strong promoters. The plasmid was linearized and randomly integrated into the genome of *Y. lipolytica* by the NHEJ-mediated method, and a gene overexpression library was thus constructed. We screened the resulting strains by shake-flask fermentation. As expected, the strains exhibited variations in growth and MVA accumulation (Fig. 2a). These differences should be caused by variations in the genomic location of the inserted DNA, which results in variable gene expression. The highest production of MVA reached 1.96 g/L at 96 h, and this strain was named AHH12 (Fig. 2a). Accumulation of MVA was not detected in control strain PO1f (Fig. 2b). Compared with PO1f, AHH12 showed slightly faster growth (Fig. 2c). We used strain AHH12 as a starter strain to construct an α-farnesene biosynthesis pathway. AHH12 can also be used as a platform for production of other terpenoids.

### Construction of α-farnesene biosynthesis pathway

**Fig. 3:**
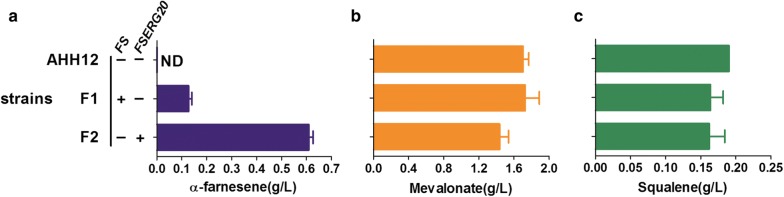
α-Farnesene, mevalonate and squalene production in a strain expressing the α-farnesene synthesis pathway in *Y. lipolytica*. **a** α-Farnesene titer. **b** Mevalonate accumulation. **c** Byproduct squalene accumulation. All data were detected after 120 h of fermentation in 300-mL shaken flasks containing 50 mL YPD medium and represent the mean ± SD of biological triplicates

To synthesize α-farnesene in *Y. lipolytica*, we expressed the codon-optimized gene encoding α-farnesene synthase (FS) from apple seeds following NHEJ-mediated genome integration. The strain F1 with the highest production of α-farnesene was obtained from screening different transformants, and it produced 0.13 g/L α-farnesene (Fig. 3a). There was no significant difference in growth rate and glucose consumption between strains F1 and AHH12 (Additional file 1: Fig. S1). Previous study reported that 259.98 mg/L α-farnesene was accumulated in *Y. lipolytica*, which overexpressed α-farnesene synthase gene from the same resource though the pINA1312 gene expression plasmid [30]. Compared with it, the random gene integration mediated by NHEJ and genes overexpression library screening showed a great advantage. Interestingly, we detected MVA accumulation of 1.73 g/L for strain F1, which did not change significantly compared with that for strain AHH12 (1.70 g/L). This indicated that only a very small portion of MVA was directed to the synthesis of α-farnesene. To direct more flux to the synthesis of α-farnesene, ERG20 was fused with FS via a GGGS amino acid linker (generating FSERG20) and overexpressed to channel the intermediate FPP to α-farnesene synthesis. The fusion is conducive to improve the catalytic efficiency of FS to FPP, and avoid FPP to branch metabolism pathways, such as farnesol and squalene synthesis [30, 34]. The expression of FSERG20 improved α-farnesene titer by 4.8-fold; the titer reached 0.61 g/L in strain F2 (Fig. 3a). Consistent with the increased α-farnesene production, the accumulation of MVA decreased by 16% (Fig. 3b). The growth rate and glucose consumption rate of strain F2 did not change significantly compared with those of strain AHH12 and F1 (Additional file 1: Fig. S1). Squalene is derived from FPP by squalene synthase, and it can be used as an indicator of the flux of isoprene intermediates through the sterol pathway [35]. We found that squalene accumulation was slightly lower in strains F1 and F2 than in AHH12 (Fig. 3c), suggesting that the metabolic flux of FPP to the sterol pathway was slightly lower in the α-farnesene producing strains. Although α-farnesene production increased, high MVA accumulation was still observed in the fermentation broth. Therefore, it is necessary to further increase the flux from MVA to FPP.

### Increasing flux to α-farnesene production through overexpression of MVA pathway genes

**Fig. 4:**
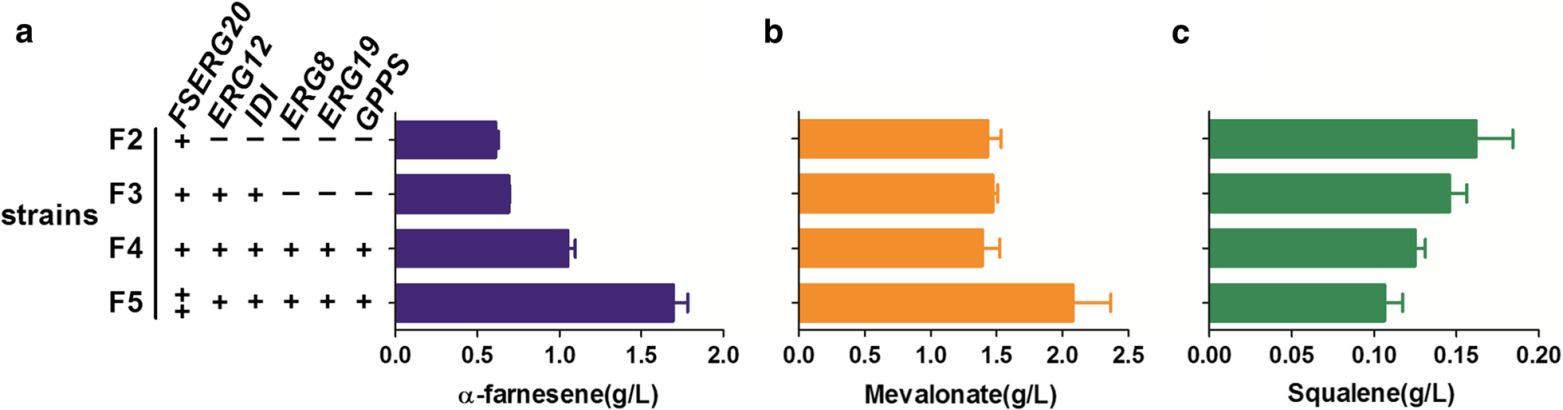
α-Farnesene, mevalonate and squalene production in a strain overexpressing endogenous genes of the mevalonate pathway (the second module in Fig. 1). **a** α-Farnesene production. **b** Mevalonate accumulation. **c** Byproduct squalene accumulation. All data were detected after 120 h of fermentation in 300-mL shaken flasks containing 50 mL YPD medium and represent the mean ± S.D. of biological triplicates

To enhance metabolic flux from MVA to FPP, we overexpressed mevalonate kinase-encoding gene *ERG12*, phosphomevalonate kinase gene *ERG8*, mevalonate diphosphate decarboxylase encoding gene *ERG19*, *IDI*, and geranyl diphosphate synthase encoding gene *GPPS* together with *FSERG20*. For this, we used NHEJ-mediated random integration followed by high-producer selection. We first overexpressed FSERG20 together with rate-limiting enzyme IDI, which converts IPP to dimethylallyl diphosphate and plays an important role in the distribution of geranyl diphosphate (GPP) and FPP flux, and mevalonate kinase, whose overexpression promotes phosphorylation of MVA. The resulting strain F3 accumulated 0.69 g/L of α-farnesene, 13% higher than the strain without *IDI* and *ERG12* overexpression (Fig. 4a). The remaining genes that encode the enzymes to catalyze the reactions from mevalonate to FPP, including phosphomevalonate kinase (ERG8), mevalonate diphosphate decarboxylase (ERG19) and GPP synthase (GPPS) were also overexpressed in strain F4. The production of α-farnesene further increased by 52%, reaching 1.05 g/L by strain F4 (Fig. 4a), which indicated that enhancing metabolic flux from MVA to FPP can effectively increase the production of α-farnesene. The enhancement of this metabolic pathway did not increase the accumulation of byproduct squalene (Fig. 4c). Unexpectedly, MVA accumulation by strains F3 and F4 did not decrease with the increase of α-farnesene production (Fig. 4b). We hypothesized that when FPP was consumed by the synthesis of α-farnesene, its precursor MVA was also consumed, which pulled more flux to the MVA biosynthesis pathway, and thus the overall accumulated level of MVA did not change significantly.

Considering the accumulation of MVA, which indicated that there was abundant precursor available for α-farnesene biosynthesis, further increasing the downstream metabolic strength might benefit α-farnesene production. Therefore, we constructed strains which integrated an additional copy of *FSERG20*, and transformants were selected. The strain with the highest α-farnesene production was named F5. The α-farnesene production of strain F5 increased by 65%, and the titer reached 1.70 g/L (Fig. 4a). Compared with the above strains, F5 showed lower squalene accumulation (Fig. 4c), confirming more FPP was distributed to α-farnesene synthesis. MVA production by strain F5 was higher than that by strain F4 (Fig. 4b), which was in accordance with our hypothesis above. Faster growth and glucose consumption were observed in F5 (Additional file 1: Fig. S1). Through integration of MVA pathway genes, α-farnesene production increased by 13-fold, which is much higher than that previously reported in *Y. lipolytica* [30]. These results suggest that our MVA production strain AHH12 is a very good background strain for constructing α-farnesene production strains; it could also be used as a platform for synthesis of other terpenoids. Our work also demonstrates that NHEJ-mediated gene integration is an effective method to obtain strains with high gene expression and high levels of product synthesis.

### Higher α-farnesene production through optimization of cultivation

**Fig. 5:**
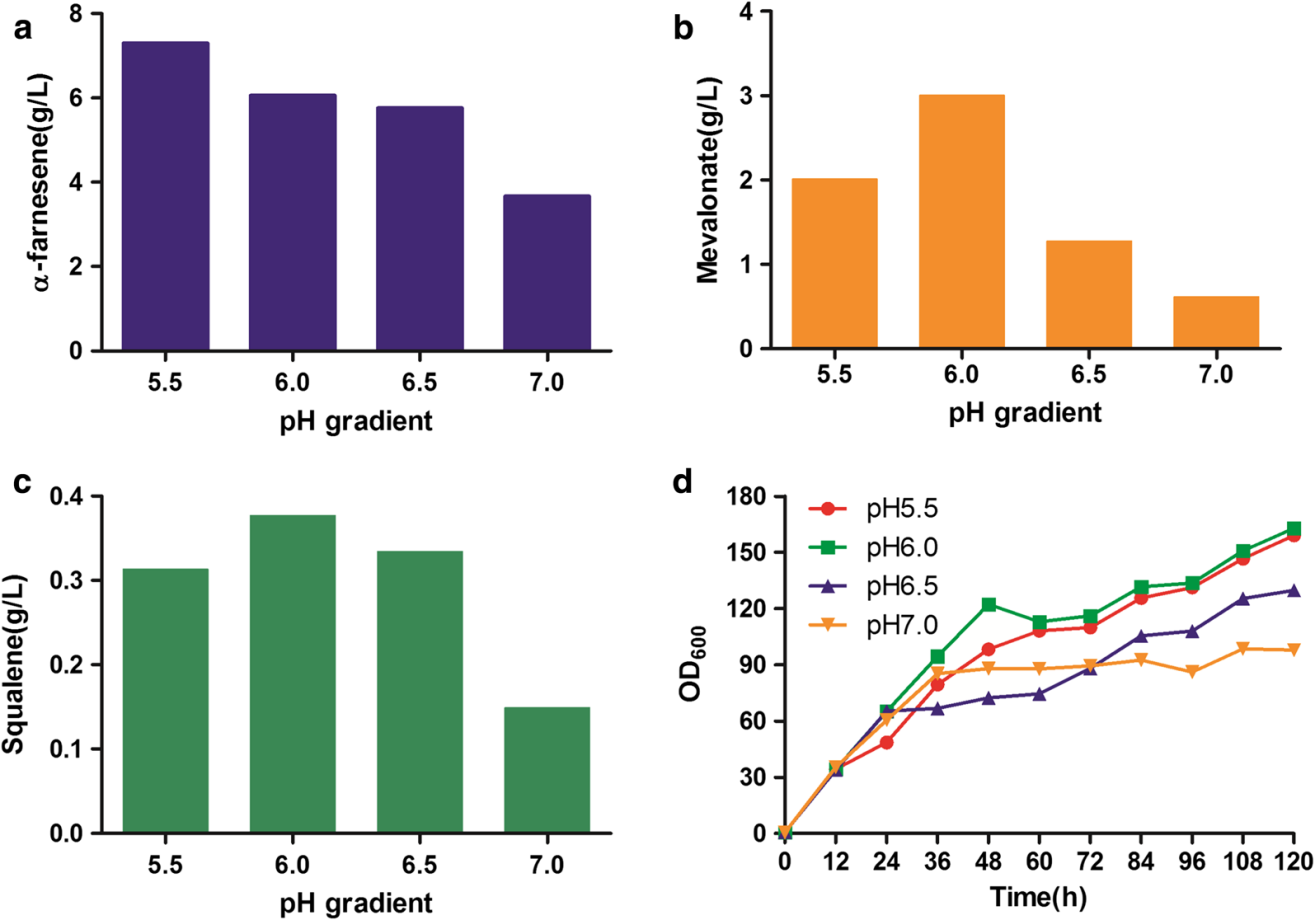
Optimization of pH for fermentation of strain F5 in bioreactor. **a** Titer of α-farnesene at different pHs. **b** Mevalonate accumulation. **c** Byproduct squalene accumulation. **d** Growth curve during fermentation. α-Farnesene, mevalonate and squalene were detected after 120 h of fermentation in a 1-L bioreactor containing 800 mL YPD medium. This experiment was performed with an *n* = 1

We optimized the cultivation conditions using the best α-farnesene producing strain, F5. We explored the effects of pH, dissolved oxygen and stirring speed on the production of α-farnesene in a 1-L fermenter. The pH of the fermentation broth was controlled at 5.5, 6.0, 6.5, or 7.0. The strain showed the highest α-farnesene accumulation to 7.3 g/L when the pH was 5.5, accompanied by the accumulation of 2.01 g/L MVA and 0.31 g/L squalene (Fig. 5). In terms of growth, the highest biomass was obtained at pH 5.5 and 6.0. *Y. lipolytica* is an acid-tolerant yeast and perhaps acidic pH is more suitable for α-farnesene synthesis and biomass production.

**Fig. 6:**
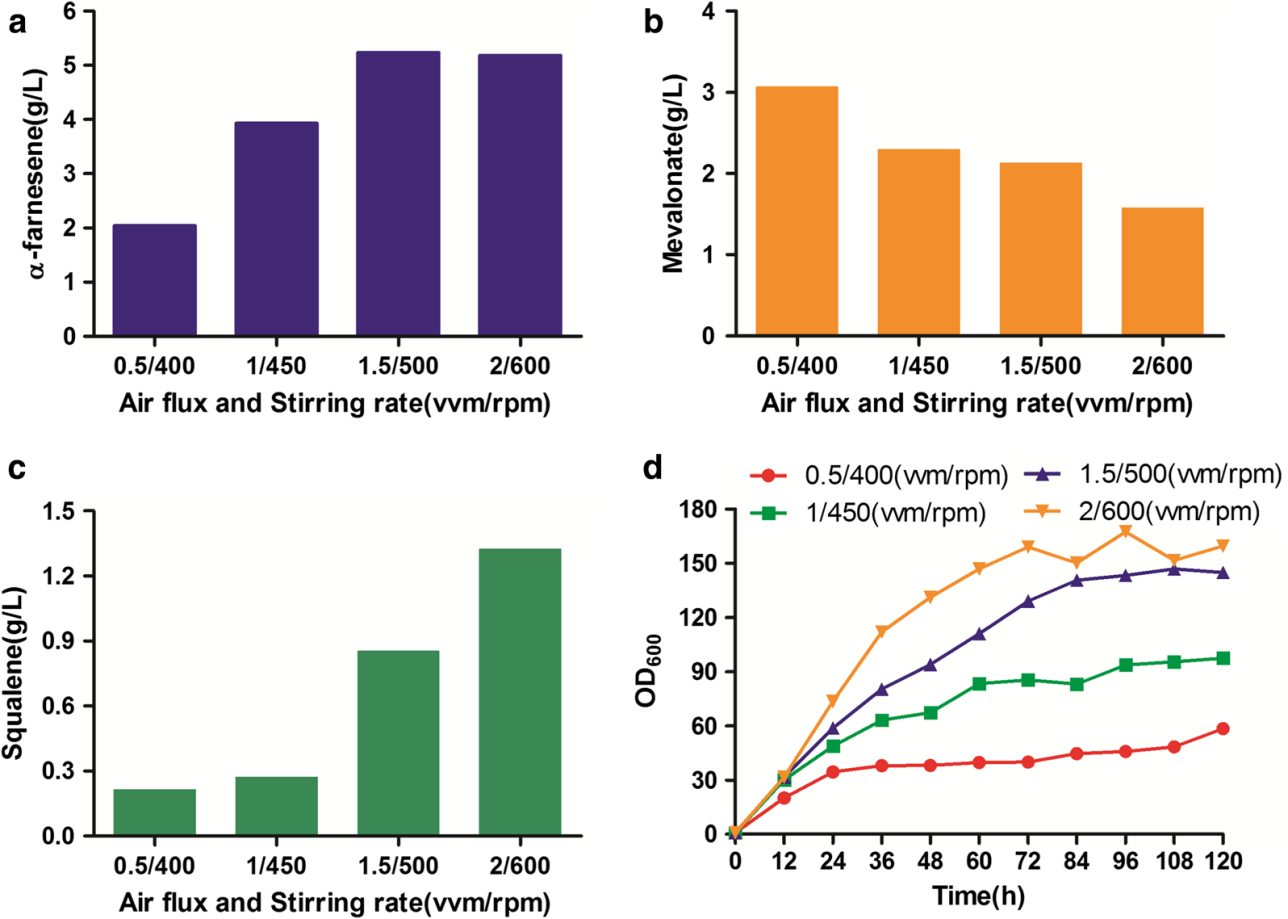
Optimization of air flux and stirring rate for fermentation of strain F5 in bioreactor. **a** Titer of α-farnesene. **b** Mevalonate accumulation. **c** Byproduct squalene accumulation. **d** Growth curve during fermentation. α-Farnesene, mevalonate and squalene were detected after 120 h of fermentation in a 1-L bioreactor containing 800 mL YPD medium. This experiment was performed with an *n* = 1

On the basis of pH 5.5, air flux and stirring rate were tested. Four combinatorial conditions were set up: 0.5/400, 1.0/450, 1.5/500, and 2.0/600 (vvm/rpm). The combination of 2 vvm and 600 rpm resulted in the fastest growth rate (Fig. 6). With increasing air flux and stirring rate, the accumulation of MVA decreased, while the production of squalene increased gradually. Interestingly, the α-farnesene titer was slightly higher at 1.5/500 than 2.0/600 (vvm/rpm) at 120 h (5.24 and 5.19 g/L α-farnesene, respectively). However, the α-farnesene yield was higher with combination 1.5/500 (0.031 g/g glucose) than 2.0/600 (0.023 g/g glucose). Therefore, 1.5 vvm and 500 rpm were the best conditions for α-farnesene production. Dissolved oxygen is important to balance the NADH/NAD^+^ ratio and the shunt ratio of carbon source metabolism, which may affect α-farnesene synthesis and biomass production [36, 37]. In short, the combination of pH 5.5 and 1.5 vvm air flux with 500 rpm stirring rate is favorable condition for α-farnesene production.

**Fig. 7:**
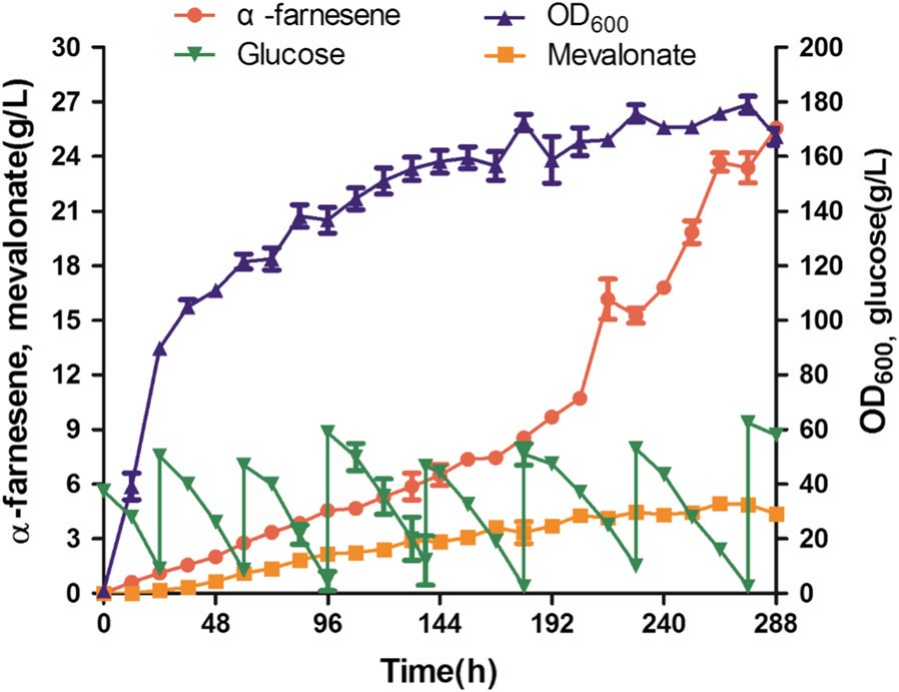
α-Farnesene and mevalonate production, glucose consumption and growth of strain F5 in fed-batch cultivation. The cultivation was carried out in a 1.0-L bioreactor containing 800 mL YPD medium at pH 5.5, 1.5 vvm air flux, and 500 rpm stirring rate. Glucose was fed when the concentration was < 10 g/L. The error bars represent standard deviations of duplicate cultivations

After determining the optimum conditions for the production of α-farnesene by strain F5, we carried out fed-batch fermentation in these conditions. During the fermentation process, the accumulation of α-farnesene increased with time and reached 25.55 g/L at 288 h, which is the highest reported terpenoid titer in *Y. lipolytica*. OD_600_ reached 170 at the end of fermentation. The production of MVA was about 4.5 g/L (Fig. 7). The final accumulation of squalene was 0.21 g/L (Additional file 1: Fig. S2). Interestingly, we detected accumulation of a large amount of citric acid (35.1 g/L) and mannitol (33.3 g/L) (Additional file 1: Fig. S2), which indicated that even though MVA pathway genes were overexpressed, some carbon flux was still directed to byproduct synthesis, resulting in waste of substrates. The accumulation of citric acid might be caused by nitrogen limitation due to the high biomass concentration [38, 39], and it indicated that the activity of citrate lyase needs to be improved to direct more citric acid into acetyl-CoA synthesis. The formation of mannitol is a result of excessive NADH accumulation. In *Y. lipolytica* and *Aspergillus niger*, when oxygen is limited, excess NADH cannot form NAD^+^ through oxidative phosphorylation, and cells accumulate mannitol to balance NADH/NAD^+^ [40, 41]. In addition, it was reported that fed-batch fermentation can promote the accumulation of mannitol [38, 42]. Our results demonstrate that optimization of fermentation can significantly improve α-farnesene production. However, in future work, further engineering strategies such as overexpression of citrate lyase and directing mannitol into the glycolysis pathway could be used to convert these byproducts into acetyl-CoA to provide more precursor for the MVA pathway.

## Conclusions

In this study, we constructed gene overexpression libraries by NHEJ-mediated genome integration, and screened strains with high production capacity for target products. Using this strategy, we first constructed a MVA production strain. Using the same approach, we then constructed strains based on this platform expressing FS and overexpressing all the genes in the MVA pathway, to enable *Y. lipolytica* to efficiently synthesize α-farnesene. Through a series of push–pull strategies, the α-farnesene production reached 1.70 g/L, improved by 13-fold compared with the strain only expressing FS. Optimization of fermentation conditions further increased the α-farnesene production to 25.55 g/L, which is the highest terpenoid titer reported in *Y. lipolytica*. In conclusion, the strategy of NHEJ-mediated genome integration and screening of the resulting gene overexpression libraries is effective to obtain strains with high gene expression and high product synthesis. Our work demonstrates that *Y. lipolytica* has great potential for terpenoid synthesis.

## Materials and methods

### Strains, media, and culture conditions

*Escherichia coli* strain DH5α was used for plasmid construction and proliferation, and was grown in Luria–Bertani (LB) complete medium (10 g/L NaCl, 10 g/L tryptone, 5 g/L yeast extract) with 20 g/L agar added to prepare solid medium. Ampicillin (50 mg/L) was added to LB when necessary. *E. coli* strains were cultured at 37 °C, 200 rpm. *Y. lipolytica* strains used in this study are listed in Table 1. YPD medium (20 g/L glucose, 20 g/L tryptone, 10 g/L yeast extract) was used for cultivation of *Y. lipolytica* strains. Synthetic dextrose (SD) medium [20 g/L glucose, 1.7 g/L yeast nitrogen base, 5 g/L (NH_4_)_2_SO_4_] supplemented with suitable amino acid dropout mixes was used for transformant selection. Hygromycin B (400 or 800 mg/L; Yeasen, Shanghai, China) and 20 g/L agar were added to YPD and SD medium when necessary. *Y. lipolytica* strains were cultured at 30 °C, 220 rpm.

**Table 1:** Strains used in this study

Name	Description	References
PO1f	*MatA*, *Leu2*-*270*, *URA3*-*302*, *xpr2*-*322*, *axp*-*2*	INRA
AHH12	PO1f with integrated fragment of linearized AtoB-HMGR-HMGS plasmid	This study
F1	AHH12 with integrated fragment of linearized plasmid pKi-1-FS	This study
F2	AHH12 with integrated fragment of linearized plasmid JMP-hyg-FSERG20	This study
F3	AHH12 with integrated fragment of linearized plasmid JMP-hyg-FSERG20-IDI-ERG12	This study
F4	AHH12 with integrated fragment of linearized plasmid JMP-hyg-FSERG20-IDI-ERG12, and 114-GPPS-ERG8-ERG19	This study
F5	AHH12 with integrated fragment of linearized plasmid JMP-hyg-FSERG20-IDI-ERG12, 114-GPPS-ERG8-ERG19, and YLEP-URA-FSERG20	This study

### Construction of plasmids and strains

The primers used for plasmid construction are listed in Additional file 1: Table S1. Primers were synthesized by TsingKe (Beijing, China). DNA fragments were obtained by polymerase chain reaction (PCR) using GoldenStar T6 DNA polymerase (TsingKe). The restriction enzymes used in this study were purchased from Thermo Fisher Scientific (Shanghai, China). All heterologous genes were optimized according to the codon preference of *Y. lipolytica* and synthesized by GENERAL BIOSYSTEMS (Anhui, China). Gibson assembly [43] was used for plasmid construction. All the plasmids used in this study are shown in Table 2.

**Table 2:** Plasmids used in this study

Name	Description	References
pKi-1	*Yarrowia lipolytica* integrative vector, *ut8* promoter with *CYC1* terminator, *Leu2* selection marker	[28]
pKi-2	*Yarrowia lipolytica* integrative vector, *hp4d* promoter with *XPR2* terminator, *TEF1* promoter with *CYC1* terminator, *URA3* selection marker	[28]
JMP-hyg	*Yarrowia lipolytica* integrative vector, *GPD* promoter with *XPR2* terminator, Hygromycin B selection marker	[28]
114-EXP-FBA	*Yarrowia lipolytica* integrative vector, *EXP* promoter with *ICL* terminator, *FBA* promoter with *LIP2* terminator, *Leu2* selection marker	[28]
YLEP-URA	*Yarrowia lipolytica* episomal vector, *ut8* promoter with *CYC1* terminator, *URA3* selection marker	[28]
pki-AtoB-HMGR-HMGS	pKi-2 vector containing codon-optimized *AtoB* under *TEF1* promoter, *HMGS* under *hp4d* promoter, and *ut8*-*HMGR* (codon-optimized) -*CYC1* fragments	This study
pKi-1-FS	pKi-1 vector containing codon-optimized *FS*	This study
JMP-hyg-FSERG20	JMP-hyg vector containing a fusion gene of codon-optimized *FS* and *ERG20* with GGGS linker between *FS* and *ERG20* (*FSERG20*)	This study
JMP-hyg-FSERG20-IDI-ERG12	JMP-hyg-FSERG20 plasmid containing *TEF1*-*ERG12*-*CYC1* and *GPD*-*IDI*-*XPR2* fragments	This study
114-GPPS-ERG8-ERG19	114-EXP-FBA vector containing *GPPS*, *GPD*-*ERG8*-*XPR2* and *TEF1*-*ERG19*-*CYC1* fragments	This study
YLEP-URA-FSERG20	YLEP-URA vector containing *FSERG20*	This study

To construct a high MVA titer strain, plasmid pki-AtoB-HMGR-HMGS was constructed based on vector pki-2. *AtoB* (GenBank: b2224) from *E. coli* was placed under the control of the *TEF1* promoter, the HMG-CoA synthase gene (*HMGS*, GenBank: YALI0_F30481g) from *Y. lipolytica* was placed under the control of the *hp4d* promoter, and the HMG-CoA reductase gene (*HMGR*, GenBank: Bpet3342) from *B. petrii*, combined with the *ut8* promoter and *CYC1* terminator by overlap PCR, was inserted into the NheI restriction site of pki-2. The final recombinant plasmid was named pki-AtoB-HMGR-HMGS. The plasmid was linearized by SspI, transformed into *Y. lipolytica* strain PO1f, and integrated into the genome via NHEJ-mediated integration. Transformants were selected on SD-URA plates and were checked by clone PCR. The transformants were screened by fermentation in shaken flasks, and the strain with the highest MVA titer was named AHH12. The *URA* marker of AHH12 was removed using the Cre–Loxp system [44].

The α-farnesene synthase gene (*FS*, GenBank: NM_001293893) from apple seeds was codon optimized and synthesized. *FS* without a stop codon was fused with an FPP synthase gene (*ERG20*, GenBank: YALI0_E05753g) via a GGGS amino acid linker to construct *FSERG20*. *FS* and *FSERG20* were inserted into plasmids pki-1 and JMP-hyg, respectively, forming pki-1-FS and JMP-hyg-FSERG20. The two plasmids were linearized by SspI and transformed into strain AHH12, respectively. The strains with the highest α-farnesene titer, named F1 and F2, respectively, were screened by the same method as strain AHH12. The endogenous IPP isomerase gene (*IDI*, GenBank: YALI0_F04015g) with the *GPD* promoter and *XPR2* terminator and the mevalonate kinase gene (*ERG12*, GenBank: YALI0_B16038g) with the *TEF1* promoter and *CYC1* terminator were inserted into plasmid JMP-hyg-FSERG20 to construct JMP-hyg-FSERG20-IDI-ERG12. Strain F3 containing plasmid JMP-hyg-FSERG20-IDI-ERG12 was derived from strain AHH12 using the same method as described above. The other endogenous genes of the MVA pathway, including the phosphomevalonate kinase gene (*ERG8*, GenBank: YALI0_E06193g), mevalonate diphosphate decarboxylase gene (*ERG19*, GenBank: YALI0_F05632g), and GPP synthase gene (*GPPS*, GenBank: YALI0_D17050g), were inserted into plasmid JMP-114 to construct 114-GPPS-ERG8-ERG19. Strain F4 was obtained by transforming linearized plasmids 114-GPPS-ERG8-ERG19 and JMP-hyg-FSERG20-IDI-ERG12 into strain AHH12 and selection by detecting the α-farnesene titer. To obtain strain F5, we constructed a plasmid containing *FSERG20* with a *URA* selection marker, and named it YLEP-URA-FSERG20. This plasmid was co-transformed with 114-GPPS-ERG8-ERG19 and JMP-hyg-FSERG20-IDI-ERG12 into strain AHH12 to generate strain F5. NHEJ-mediated nucleotide insertion was used to integrate all the heterologous DNA fragments into the AHH12 genome. To screen strains F1 to F5 (screening for high α-farnesene production), we randomly selected about 20–30 transformants from each library, extracted the genomic DNA, and confirmed that every overexpressed gene was inserted into the genome by PCR. DNA fragments were transformed into *Y. lipolytica* using the lithium acetate method [45].

### Analysis of mevalonate, citric acid and mannitol

Fermentation broth (1 mL) was taken and centrifuged for 2 min at 13,000 rpm to remove cell debris, and subsequently filtered using a 0.22-μm filter, in preparation for metabolite analysis. A high-performance liquid chromatography system equipped with an Aminex HPX-87H column (BioRad, Inc., Hercules, CA) and a refractive index detector was used for analysis of MVA, citrate and mannitol. H_2_SO_4_ (5 mM) was used as the mobile phase with flow rate 0.6 mL/min at 65 °C.

### Analysis of glucose and OD_600_

The glucose concentration in medium was determined using an SBA-40D biosensor (Biology Institute of Shandong Academy of Science, Shandong, China) according to the instruction manual. A UV-1800 spectrophotometer (Shimadzu, Japan) was used to detect the absorbance of fermentation broth at 600 nm.

### Analysis of α-farnesene

Dodecane was added into medium to capture α-farnesene before culturing and 200 μL dodecane was taken from medium for α-farnesene content analysis every 12 h during fermentation. The concentration of α-farnesene was analyzed using gas chromatography (GC; Agilent Technologies, Santa Clara, CA) with an Rtx-5 capillary column (30.0 m, 0.25 mm ID, 0.25 μm df; RESTEK, USA) and a flame ionization detector (FID). The temperatures of the injector and the detector were set at 280 and 290 °C, respectively. The oven temperature was kept at 80 °C for 1 min, and then ramped to 250 °C at 10 °C/min and held for 1 min, then to 280 °C at 10 °C/min and held for 2 min.

### Analysis of squalene

Fermentation broth (2 mL) was taken and centrifuged to collect cells, which were washed once with sterile water. The cells were suspended in 400 μL KOH-ethanol solution and boiled for 5 min. After cooling to room temperature, 400 μL of dodecane were added and the mixture was vortexed for 5 min. After centrifugation, 200 μL of dodecane were taken to detect the squalene content. Samples were analyzed using the GC-FID system with Rtx-5 capillary column. The temperature of the injector and FID were set at 250 °C and 280 °C, respectively. The oven temperature was kept at 80 °C for 1 min, then ramped to 250 °C at 20 °C/min and held for 15 min.

### Optimization of fermentation in a bioreactor

Strain F5 was inoculated into a 300-mL flask containing 50 mL liquid YPD medium and cultured at 30 °C, 220 rpm, for 24 h. Then 1 mL of this culture was transferred to a 500-mL flask containing 100 mL YPD liquid medium for 24 h in the same culture conditions. The second pre-culture was transferred to a 1-L bioreactor (INFORS Multifors Bacteria, Switzerland) with 10% inoculum. Modified YPD medium (50 g/L glucose, 20 g/L tryptone, 10 g/L yeast extract) was used for fermentation, and 10% dodecane was added to the fermentation broth. Different cultivation conditions were tested, including variation of pH and various combinations of agitation speed and ventilatory capacity. The pH was set at 5.5, 6.0, 6.5, or 7.0. The air flux and stirring rate were set at 0.5/400, 1.0/500, 1.5/550, or 2.0/600 (vvm/rpm). During fermentation, feed solution containing 500 g/L glucose was added when the glucose concentration in the fermentation broth fell below 10 g/L.

## Supplementary information

**Additional file 1.** Additional figures and tables.

## Data Availability

The datasets used and analyzed during the current study are available from the corresponding author on reasonable request.

## Abbreviations

HR: homologous recombination
NHEJ: non-homologous end-joining
Acetyl-CoA: acetyl coenzyme A
MVA: mevalonate
FPP: farnesyl pyrophosphate
IPP: isopentenyl diphosphate
GPP: geranyl diphosphate
NADH: nicotinamide adenine dinucleotide
NADPH: reduced nicotinamide adenine dinucleotide phosphate
HMGR: HMG-CoA reductase
HMGS: HMG-CoA synthase
ERG10: acetyl-CoA acetyltransferase/acetyl-CoA thiolase
AtoB: acetyl-CoA acetyltransferase
ERG12: mevalonate kinase
ERG8: phosphomevalonate kinase
ERG19: mevalonate diphosphate decarboxylase
IDI: IPP isomerase
GPPS: GPP synthase
ERG20: FPP synthase
FS: codon-optimized α-farnesene synthase
FSERG20: fused FS and ERG20 with GGGS amino acid linker
SD: synthetic dextrose
PCR: polymerase chain reaction
GC: gas chromatography
FID: flame ionization detector
OD_600_: optical density at 600 nm
