# Supplementary material for: Engineering the oleaginous yeast Yarrowia lipolytica for production of α-farnesene
Source: Biotechnol Biofuels. 2019 Dec 23;12:296. doi: 10.1186/s13068-019-1636-z (PMC6927232; doi:10.1186/s13068-019-1636-z)
Supplement: Supplementary file 1 — Additional file 1. Additional figures and tables. [file 13068_2019_1636_MOESM1_ESM.docx]

**Additional file**


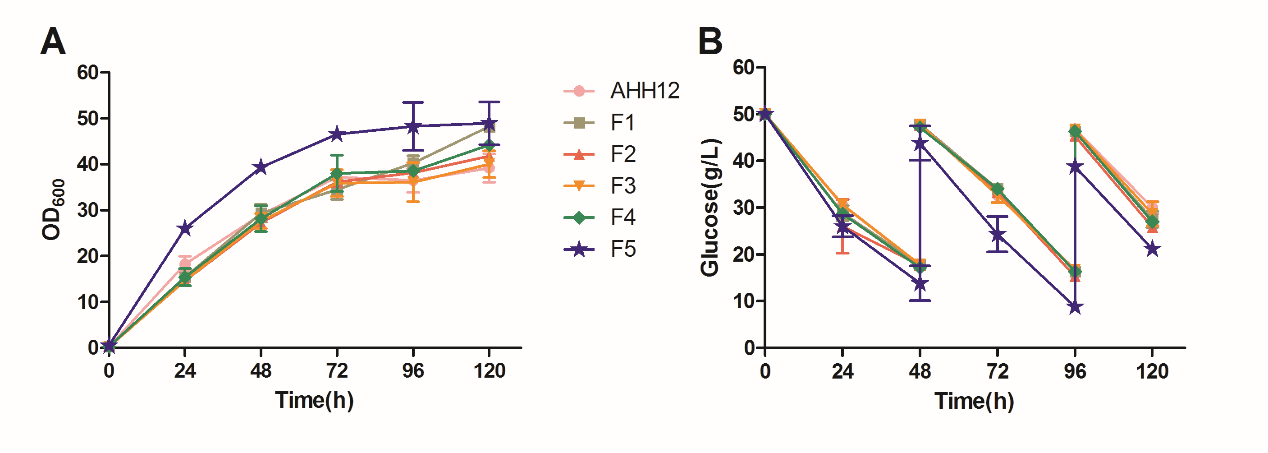


Fig. S1 Growth and glucose consumption during fermentation of constructed F1 to F5 strains. (A) Growth curve of the strains during fermentation. (B) Glucose consumption curve of the strains during fermentation. All data were detected every 24 h of fermentation in 300 mL shaking flask with 50 mL YPD medium and represent the mean±s.d. of biological triplicates.


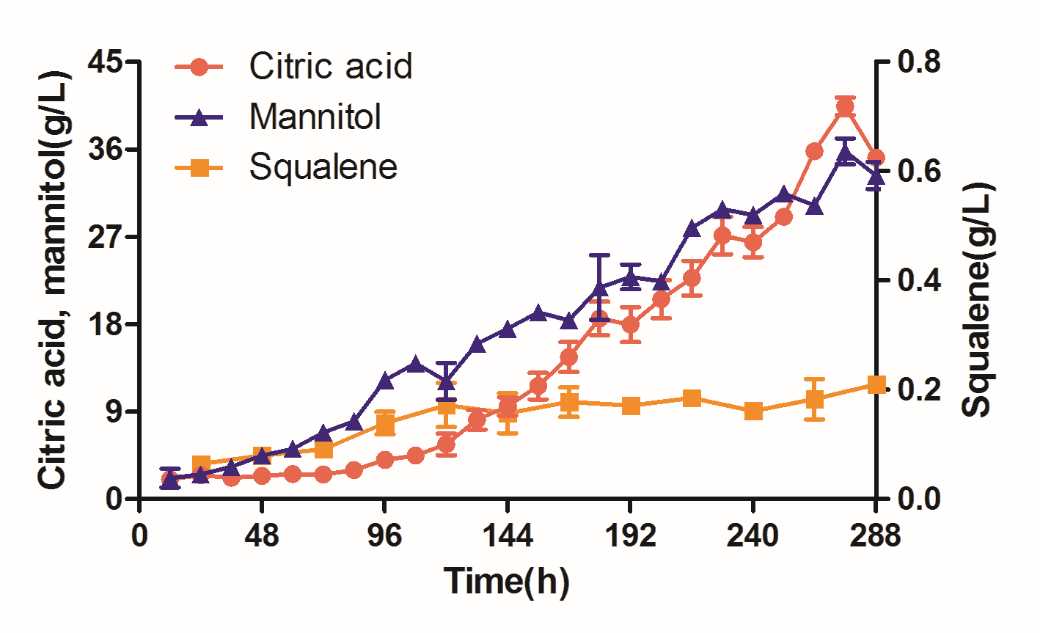


Fig. S2 Accumulation of by-products citric acid, mannitol and squalene during fermentation in 1.0 L bioreactor with 800 mL YPD medium at pH 5.5, 1.5 vvm air flux and 500 rpm stirring rate culture condition. Error bars embody standard deviation among the average of two distinct reiterated experiments.

Additional Table S1. Primers used in this study.

| Name | Sequence (5’-3’) | Purpose |
| --- | --- | --- |
| HMGS-F | ATCCACGTGGGAACCGCGATATGTCGCAACCCCAGAACGT | Amplifying *HMGS* |
| HMGS-R | AGGCCATGGAGGTACGCGATCTACTGCTTGATCTCGTACT |  |
| AtoB-F | GCAGTACTAACCGCAGATTTATGAAGAACTGTGTCATCGT | Amplifying *AtoB* |
| AtoB-R | ATAACTAATTACATGAATTTTTAGTTCAGTCGCTCAATGA |  |
| FS-F | ATAAGAATCATTCAAAGGTT ATGGAATTCCGAGTGCACCT | Amplifying *FS* |
| FS-R | ACATAACTAATTACATGATT TTAGTTCACCAGAGGCTGGA |  |
| FSERG20-F | AATTAAACACACATCAACAGATGGAATTCCGAGTGCACCT | Amplifying *FSERG20* |
| FSERG20-R | GGACAGGCCATGGAGGTACGCTACTTCTGTCGCTTGTAAA |  |
| ut8-HMGR-CYC1-F | GCCGCCAACCCGGTCTCTAAGCTAGCGGTACCAAGGAAGCATGCGGTAC | Amplifying *ut8-HMGR-CYC1* fragment |
| ut8-HMGR-CYC1-R | CACCTCAGCATGCACGCGTATCGATAAGCAAATTAAAGCCTTCGAGCGTC |  |
| IDI-F | AATTAAACACACATCAACAGATGACGACGTCTTACAGCGA | Amplifying *IDI* |
| IDI-R | GGACAGGCCATGGAGGTACGCTACTTGATCCACCGCCGAA |  |
| ERG12-F | GCAGTACTAACCGCAGATTTATGGACTACATCATTTCGGC | Amplifying *ERG12* |
| ERG12-R | ATAACTAATTACATGAATTTCTAATGGGTCCAGGGACCGA |  |
| GPPS-F | AAGACATATCTACAGCATTTATGGATTATAACAGCGCGGA | Amplifying *GPPS* |
| GPPS-R | TTGCTAAACAAACTGCATTTTCACTGCGCATCCTCAAAGT |  |
| ERG8-F | AATTAAACACACATCAACAGATGACCACCTATTCGGCTCC | Amplifying *ERG8* |
| ERG8-R | GGACAGGCCATGGAGGTACGCTACTTGAACCCCTTCTCGA |  |
| ERG19-F | GCAGTACTAACCGCAGATTT ATGATCCACCAGGCCTCCAC | Amplifying *ERG19* |
| ERG19-R | ATAACTAATTACATGAATTTCTACTTGCTGTTCTTCAGAG |  |
